# VIRsiRNApred: a web server for predicting inhibition efficacy of siRNAs targeting human viruses

**DOI:** 10.1186/1479-5876-11-305

**Published:** 2013-12-11

**Authors:** Abid Qureshi, Nishant Thakur, Manoj Kumar

**Affiliations:** 1 Bioinformatics Centre, Institute of Microbial Technology, Council of Scientific and Industrial Research, Sector 39-A, Chandigarh 160036, India

**Keywords:** Virus, siRNA, Efficacy, Prediction algorithm, RNAi

## Abstract

**Background:**

Selection of effective viral siRNA is an indispensable step in the development of siRNA based antiviral therapeutics. Despite immense potential, a viral siRNA efficacy prediction algorithm is still not available. Moreover, performances of the existing general mammalian siRNA efficacy predictors are not satisfactory for viral siRNAs. Therefore, we have developed “VIRsiRNApred” a support vector machine (SVM) based method for predicting the efficacy of viral siRNA.

**Methods:**

In the present study, we have employed a new dataset of 1725 viral siRNAs with experimentally verified quantitative efficacies tested under heterogeneous experimental conditions and targeting as many as 37 important human viruses including HIV, Influenza, HCV, HBV, SARS etc. These siRNAs were divided into training (T^1380^) and validation (V^345^) datasets. Important siRNA sequence features including mono to penta nucleotide frequencies, binary pattern, thermodynamic properties and secondary structure were employed for model development.

**Results:**

During 10-fold cross validation on T^1380^ using hybrid approach, we achieved a maximum Pearson Correlation Coefficient (PCC) of 0.55 between predicted and actual efficacy of viral siRNAs. On V^345^ independent dataset, our best model achieved a maximum correlation of 0.50 while existing general siRNA prediction methods showed PCC from 0.05 to 0.18. However, using leave one out cross validation PCC was improved to 0.58 and 0.55 on training and validation datasets respectively. SVM performed better than other machine learning techniques used like ANN, KNN and REP Tree.

**Conclusion:**

VIRsiRNApred is the first algorithm for predicting inhibition efficacy of viral siRNAs which is developed using experimentally verified viral siRNAs. We hope this algorithm would be useful in predicting highly potent viral siRNA to aid siRNA based antiviral therapeutics development. The web server is freely available at http://crdd.osdd.net/servers/virsirnapred/.

## Background

Viruses like Influenza, Hepatitis, Dengue, SARS coronavirus (SARS-CoV), Human Immunodeficiency Virus (HIV) etc. remain a public health concern worldwide due to their emerging and re-emerging nature [1]. Due to lack of therapeutics against majority of viruses, there is always a need to develop more effective antiviral agents [2]. Lately, RNA interference (RNAi) has emerged as a potential therapeutic tool for targeting human viruses. RNAi or gene silencing is a process by which sequence-specific degradation of mRNA takes place [3]. In this process, long dsRNA precursors are chopped into shorter (19–23 resides) units by a ribonuclease enzyme called dicer. These short interfering RNAs (siRNAs) possess two terminal nucleotide 3′ overhangs. After that a ribonucleoprotein machinery called RNA induced silencing complex (RISC) incorporates one of the siRNA strands and cleaves the complementary target mRNA using ATP [4].

Researchers have extensively used RNAi process to target a number of viral genes to suppress their expression [5,6]. siRNAs targeting different regions of the HIV genome in infected cells showed promising results in inhibiting viral replication [7,8]. Also, siRNAs targeting the influenza virus nucleocapsid and RNA transcriptase genes restricted its transcription and replication [9,10]. Similarly, siRNAs directed against the Hepatitis B virus surface regions prevented the virus production [11]. siRNAs employed against SARS-CoV envelope and genes were able to effectively block their expression [12]. In another study, siRNAs targeting Dengue virus genes were able to impede the viral infection [13]. In addition, siRNAs have been shown to curb many other viruses like Human papillomavirus (HPV) [14], West Nile virus (WNV) [15] etc.

RNAi methodology has many desirable features to use as antiviral agents. It can target diverse types of viral genomes, whether it be double/single stranded DNA or RNA, which make it a suitable candidate for broad-spectrum antiviral therapy [16]. Also, siRNA aims at small length of the target mRNA instead of a functional domain of a protein, therefore, even a small viral genome can lend many targetable regions [5]. Further, many siRNAs may be expressed simultaneously to increase inhibition in a coordinated manner [17]. They are also harnessed to degrade mRNAs which generate disease causing proteins [6]. siRNA based drugs have also entered the clinical trials for various human diseases e.g. kidney disorders, LDL lowering, ocular/retinal disorders, cancer and viral diseases [18]. Specifically for Respiratory syncytial virus (RSV), ALN-RSV01 has completed Phase II trial. Yet another drug named SPC3649, developed by Santaris Pharma targets Hepatitis C virus (HCV) and was also under Phase II clinical trial [19,20]. Many researchers have further reviewed the importance of RNAi in inhibiting viral infections [20,21].

To predict effective siRNAs, a number of general siRNA design rules as well prediction algorithms been developed since every siRNA designed against a given mRNA are not equally effective [22,23]. The earliest guidelines for siRNA design were based on presence and/or absence of specific nucleotide residues at different positions in siRNA as proposed by Elbashir [24], Reynolds [23], Ui-Tei [25], Amarzguioui [26] and Jagla [27]. Naito et al. have used the guidelines of Ui-Tei, Reynolds and Amarzguioui to select effective siRNAs against viruses [28].

Subsequently, machine learning techniques like SVM, ANN etc. have been utilized to predict effective mammalian siRNAs [29,30] or their efficacy [31,32]. Performance of these methods was better than the general siRNA design rules [33]. Saetrom used boosted genetic programming to predict mammalian siRNA efficacy on a modest dataset of 581 siRNAs [34]. Later Huesken et al. reported the screen method of functional siRNAs by using an artificial neural network [35]. Holen reported siRNA rules based on apparent overrepresentation or underrepresentation of certain nucleotides at different positions [36]. Researchers have utilised many siRNA features like nucleotide composition [37], thermodynamic parameters [38], nucleotide position [39] etc. to predict the efficiency of siRNAs. Many other workers have also used a combination of features using different machine learning techniques to predict mammalian siRNA efficacy [40-42].

When we used best of these existing algorithms for prediction of viral siRNA, their performance was far from satisfactory. It could be because none of these methods were developed using virus specific siRNAs. Therefore, this prompted us to make virus specific siRNA efficacy prediction algorithm using experimentally validated viral siRNA dataset. Here we employed machine learning techniques to predict the inhibition efficacy of siRNAs. This will help the researchers to select the best siRNAs for use as potential therapeutics against important human viruses.

## Methods

### Algorithm development

#### Data collection

We have chosen our dataset from Viral siRNA database (VIRsiRNAdb) which contains over 1358 siRNA sequences targeting different human viruses and HIV siRNA database (HIVsirDB) having over 750 entries. From these databases we selected 927 and 240 sequences of 19mer which were having numerical (quantitative) efficacy. In addition 1204 sequences from patents were also used. In addition 67 more siRNAs were collected from the literature. This combined dataset of 2294 siRNAs was reduced to **1725** sequences after removal of redundant sequences. From this dataset we randomly selected 345 sequences for validation (**V**^**345**^) and the rest 1380 sequences were used in training (**T**^**1380**^).

#### siRNA sequence features

In total 12 different parameters were employed including four siRNA sequence features as nucleotide frequencies, binary pattern, thermodynamic properties, secondary structure features and their combinations or hybrids.

1. Nucleotide Frequencies

In the past many scientists have utilised nucleotide composition to predict siRNA potency. [31,40,42]. Nucleotide frequency is the number of each nucleotide in a siRNA. Since the length of our siRNAs was constant, we used nucleotide frequency instead of composition. The objective of calculating nucleotide frequencies of siRNA sequences is to transform any length of nucleotide sequence to fixed length feature vectors. This is important while using machine-learning techniques because it requires fixed length pattern. The information of each siRNA can be encapsulated to a vector of 4, 16, 64, 256, 1024 etc. multi-dimensions using frequencies of its mononucleotide, dinucleotide, triinucleotide, tetranucleotide and pentanucleotide sub sequences respectively.

2. Binary Pattern

Previous reports have used nucleotide positions to predict the RNAi activity of siRNAs [31,39]. We employed binary pattern to extract siRNA features based on the occupancy of nucleotides at each position of siRNA sequences. Four binary patterns used for each nucleotide are follows A = 1000, C = 0100, G = 0010, U = 0001 and this resulted in generation of 76 patterns for each 19-mer siRNA.

3. Thermodynamic Properties

Thermodynamic stability of the siRNA has been considered by previous workers as an important feature in siRNA design [37]. They were calculated by the method of Khvorova et al. [43]. The thermodynamic dimensions correspond to the Gibbs free energy stability of the nucleotide pairs of the siRNAs. In total 21 features were used to calculate the energies of the different sets of interacting nucleotides. These features include the binding free energies and stabilities of the folded structures.

4. Secondary Structure

Secondary structure represents the capability of a single molecule of nucleic acid sequence to form intra molecular contacts, thereby stabilizing certain sequence parts as double stranded. Secondary structure was calculated using RNAfold programme of Vienna RNA package as implemented by Peek [31]. It predicts the minimum free energy (MFE) secondary structure and equilibrium base-pairing probabilities of single sequences [44]. The secondary structures predicted by RNAfold are depicted as brackets and dots indicating ‘paired’ and ‘unpaired’ nucleotides respectively. These categorical attributes are made readable to SVM by converting them into numeric data representing the structural features as vectors.

5. Hybrid Approaches

In hybrid approach, besides the features being used individually, combinations of siRNA sequence parameters were used in order to increase the performance of the prediction method as done by earlier researchers [27,42]. We have used four hybrid methods notably mono-di-tri-tetra-penta-binary which makes a vector of total 1440 and mono-di-tri-tetra-penta-binary-thermo which makes a vector of total 1489 features.

6. Leave one out cross validation (LOOCV)

In this method each siRNA is kept for testing iteratively while remaining viral siRNAs are used for training the respective predictive models. Besides LOOCV, we have also carried out Leave one virus out cross validation (LOVOCV) strategy. In this approach, siRNAs from each virus are iteratively excluded and SVM is trained on the remaining virus siRNAs followed by testing on the excluded siRNAs of that individual virus.

7. Viral siRNA target conservation

Target site conservation analysis of all experimental 1725 viral siRNA sequences was done by matching each siRNA sequence with its respective reference viral genome sequences taken from NCBI. For this purpose, we have used ALIGN0 algorithm [45], which computes the alignment of two DNA sequences without penalizing for end-gaps.

### Algorithm and server implementation

Support vector machines (SVMs) were trained with the selected sequence features to predict siRNA potency. SVM allows choosing a number of parameters and kernels The *SVMlight* software package (available at http://svmlight.joachims.org/) was used to construct SVM classifiers. In this study, we used the radial basis function (RBF) kernel:

$$k\left(\bar{x},\bar{y}\right)=\text{exp}\left(‒\gamma ǁ\;\bar{x}‒{\bar{y}ǁ}^{2}\right)$$

where x̅ and y̅ are two data vectors, and γ is a training parameter.

Artificial Neural Network (ANN) was implemented using Stuttgart Neural Network Simulator (SNNS) package (available at http://www.ra.cs.uni-tuebingen.de/SNNS/) while K-Nearest Neighbour (KNN) and Reduced Error Pruning (REP) Tree algorithms were implemented using Weka machine learning software suite (available at http://www.cs.waikato.ac.nz/ml/weka/).

The server is implemented on Red Hat Linux and Apache (2.2.17) in back-end and front-end of web interface is implemented with PHP (5.2.14).

### Validation

In order to evaluate performance of our models, we used Pearson’s correlation coefficient (R). All models were evaluated using ten-fold cross validation technique.

$$R=\frac{n\sum_{n=1}^{n}E_{i}^{\text{act}}E_{i}^{\text{pred}}‒\sum_{n=1}^{n}E_{i}^{\text{act}}\sum_{n=1}^{n}E_{i}^{\text{pred}}}{\sqrt{n\sum_{n=1}^{n}{\left(E_{i}^{\text{act}}\right)}^{2}‒{\left(\sum_{n=1}^{n}E_{i}^{\text{act}}\right)}^{2}}\sqrt{n\sum_{n=1}^{n}{\left(E_{i}^{\text{pred}}\right)}^{2}‒{\left(\sum_{n=1}^{n}E_{i}^{\text{pred}}\right)}^{2}}}$$

Where n is the size of test set, E_i_^pred^ and E_i_^act^ is the predicted and actual efficacy respectively.

## Results

### Performance evaluation of predictive models during 10-fold cross validation

Viral siRNA prediction modelshave been developed using various siRNA sequence features including mono, di, tri, tetra, penta nucleotide frequencies, binary pattern of nucleotides, thermodynamic properties and secondary structure. During 10-fold cross validation using SVM we achieved a maximum correlation of 0.19, 0.32, 0.42, 0.43, 0.46, 0.19, 0.26, 0.07 respectively for the above-mentioned individual features, between predicted and actual efficacy of viral siRNAs. We employed different features like nucleotide frequency (mono to penta) and positional (binary) as well as structural and thermodynamic features on viral siRNA training dataset consisting of 1380 (T^1380^) sequences. During 10-fold cross validation using SVM, the Pearson Correlation Coefficient (PCC) increased from 0.19 to 0.46 while moving from individual mono to pentanucleotide frequency features respectively. The binary feature performed similar to mononucleotide frequency. The thermodynamic properties showed marginal correlations with siRNA inhibition while secondary structural features did not work. Also, we found that hybrid models (frequency-binary and frequency-binary-thermo) performed better compared to other features used individually. Amongst the hybrid models, PCC was found to increase by combining frequency, binary and thermodynamic feature vectors (0.55) as compared to hybrid frequency (0.48), hybrid frequency-binary (0.50) or hybrid frequency-binary-thermo-secondary structural features (0.53) as shown in Table 1.

**Table 1:** **Ten-fold cross validation performance of predictive models on viral siRNA dataset of 1380 sequences (T**^**1380**^**) using SVM, ANN, KNN and REP Tree machine learning techniques**

**Predictive model no.**	**siRNA features**		**No. of siRNA features**	Pearson correlation coefficient* on training (T^**1380**^**) dataset# during 10-fold cross validation**			
				**SVM**	**ANN**	**KNN**	**REP Tree**
**1**		Mononucleotide frequency	4	0.19	0.10	0.11	0.10
**2**		Dinucleotide frequency	16	0.32	0.29	0.29	0.29
**3**		Trinucleotide frequency	64	0.42	0.28	0.30	0.28
**4**		Tetranucleotide frequency	256	0.43	0.28	0.30	0.30
**5**		Pentanucleotide frequency	1024	0.46	0.29	0.30	0.30
**6**		Binary	76	0.19	0.10	0.11	0.11
**7**		Thermodynamic features	21	0.26	0.22	0.21	0.20
**8**		Secondary structure	28	0.07	0.04	0.04	0.04
**9**		1 + 2 + 3 + 4 + 5	1364	0.48	0.30	0.31	0.31
**10**		6 + 9	1440	0.50	0.36	0.41	0.32
**11**		**6 + 7 + 9**	**1461**	**0.55**	**0.46**	**0.48**	**0.45**
**12**		6 + 7 + 8 + 9	1489	0.53	0.42	0.44	0.42

*Pearson Correlation Coefficient (PCC) is the correlation between experimental and predicted viral siRNA efficacy.

#T^1380^ is the training dataset of experimental viral siRNA. Predictive Models 1-8 were developed on individual siRNA features while models 9-12 were based on hybrid siRNA features.

We also used other machine learning algorithms like ANN, KNN and REP Tree to check their performance on the experimental viral siRNA data using the above mentioned features. During 10-fold cross validation, PCC increased from 0.10 to a maximum of 0.30 from mono to pentanucleotide frequency in all the three cases. Here also the binary and secondary structural features did not perform well, confirming our earlier results using SVM. However while using a combination of features (frequency-binary and frequency-binary-thermo) in hybrid models, the correlation performance increased from 0.30 to 0.46 for ANN, 0.31 to 0.48 for KNN and 0.31 to 0.35 for REP Tree (Table 1). However SVM performed better compared to other machine learning algorithms in all the cases and was thus chosen for model development.

### Performance evaluation of predictive models on independent 345 viral siRNA dataset

Besides 10-fold cross validation, we also checked the performance of our algorithms on independent dataset of 345 siRNAs (V^345^) on the above models as shown in the Table 2. Here again, the binary and secondary structural features did not perform well while the model based on thermodynamic features showed a marginal correlation of 0.19. As expected, the hybrid frequency, hybrid frequency-binary and hybrid frequency-binary-thermo-secondary structural features gave a better correlation with PCC values 0.48, 0.48 and 0.45 respectively. The best performing hybrid model with frequency-binary-thermo feature vectors gave a correlation of 0.50 and thus performed better on the validation dataset. Other machine learning techniques performed in a similar trend but their correlation was less as compared to SVM. Their best correlations were achievedwhile using the hybrid frequency-binary-thermo model. The PCCs were 0.33 for ANN, 0.35 for KNN and 0.31 for REP Tree (Table 2).

**Table 2:** **Evaluation of performance of predictive models on validation dataset of 345 viral siRNAs(V**^**345**^**)**

**Predictive model no.**	**siRNA features**		**No. of siRNA features**	Pearson correlation coefficient* on validation (V^**345**^**) dataset# during 10-fold cross validation**			
				**SVM**	**ANN**	**KNN**	**REP Tree**
**1**		Mononucleotide frequency	4	0.16	0.08	0.09	0.08
**2**		Dinucleotide frequency	16	0.30	0.23	0.22	0.24
**3**		Trinucleotide frequency	64	0.39	0.25	0.24	0.26
**4**		Tetranucleotide frequency	256	0.40	0.26	0.27	0.28
**5**		Pentanucleotide frequency	1024	0.42	0.27	0.28	0.29
**6**		Binary	76	0.03	0.02	0.02	0.01
**7**		Thermodynamic features	21	0.19	0.15	0.18	0.15
**8**		Secondary structure	28	0.02	0.02	0.02	0.02
**9**		1 + 2 + 3 + 4 + 5	1364	0.48	0.32	0.34	0.30
**10**		6 + 9	1440	0.48	0.32	0.34	0.32
**11**		**6 + 7 + 9**	**1461**	**0.50**	**0.33**	**0.35**	**0.31**
**12**		6 + 7 + 8 + 9	1489	0.45	0.32	0.33	0.30

*Pearson Correlation Coefficient (PCC) is the correlation between experimental and predicted viral siRNA efficacy.

#V^345^ is the validation dataset of experimental viral siRNA not used in training. Predictive Models 1-8 were developed on individual siRNA features while models 9-12 were based on hybrid siRNA features.

### Comparison with existing siRNA prediction algorithms

We also compared our models with existing siRNA efficacy prediction servers (some servers are not currently working or have become outdated) although they are not optimized to predict viral siRNA efficacy and are rather used for general mammalian siRNA prediction. These prediction servers were mainly developed using two mammalian siRNA datasets of Saetrom (581 siRNA) [34] and Huesken (2431 siRNA) [35]. Saetrom dataset was tested under heterogeneous experimental conditions while Huesken dataset was tested under homogenous experimental conditions. On both of these datasets (19 mer), we have also developed earlier a ‘siRNApred’ algorithm with PCC of 0.56 and 0.68 respectively during 10-fold cross validation [46]. We checked performance of ‘siRNApred’ on viral siRNA datasets of V^345^, T^1380^and (V + T)^1725^ and observed PCC of 0.10, 0.16 and 0.14 respectively. Detailed comparisons with other methods are shown in Tables 3 and 4.

**Table 3:** Comparison of VIRsiRNApred with existing siRNA efficacy prediction algorithms developed using heterogeneous siRNA dataset

**S.no**	**Reference**	**Url**	**Technique**	**siRNA data set**		Pearson correlation coefficient*		
						**Train**^**1**^	**Val**^**2**^	**V**^**345**^**#**
1	[34]	NA	GPBoost, SVM	581		0.46	0.40	Server not available
2	[37]	NA	ANN	653		0.55	0.50	Server not available
3	[32]	http://biodev.extra.cea.fr/DSIR/DSIR.html	linear	653		0.48	0.44	Server not working
4	[36]	NA	linear	526		0.55	0.52	Server not available
5	[38]	http://www.med.nagoya-u.ac.jp/neurogenetics/i_Score/i_score.html	linear	419		0.51	0.44	Server not working
6	[46]	http://www.imtech.res.in/raghava/sirnapred	SVM	581		0.56	0.47	0.10
7	VIRsiRNApred	http://crdd.osdd.net/servers/virsirnapred/	SVM	1380		**0.58**	**0.55**	**0.55**

*Pearson Correlation Coefficient (PCC) is the correlation between experimental and predicted viral siRNA efficacy.

^1^Performance on n-fold training dataset of the study.

^2^Performance on validation data set of the study.

#V^345^ is the validation dataset of experimental viral siRNA. Algorithms from S.No. 1-6 used mammalian heterogeneous siRNA datasets while S.No. 7 used experimental viral siRNA dataset.

**Table 4:** Comparison of VIRsiRNApred with existing siRNA efficacy prediction methods developed using mammalian homogeneous siRNA dataset

**S.No**	**Reference**	**Technique**	**Url**	**siRNA data set**		**Pearson correlation coefficient**		
						**Train**^**1**^	**Val**^**2**^	**V**^**345**^**#**
1	[35]	ANN	http://www.biopredsi.org	2431		0.66	0.60	Server not available
2	[32]	Linear	http://biodev.extra.cea.fr/DSIR/DSIR.html	2431		0.67	0.57	Server not working
3	[47]	Rule, SVM, RFR	http://www.bioinf.seu.edu.cn/siRNA/index.htm	3589		0.85	0.59	0.12
4	[38]	Linear	http://www.med.nagoya-u.ac.jp/neurogenetics/i_Score/i_score.html	2431		0.72	NA	0.05
5	[31]	SVM	NA	2431,		0.78	0.71	Server not available
6	[39]	Linear	http://rna.chem.t.u-tokyo.ac.jp/siexplorer.htm	702		0.77	0.60	0.18
7	[46]	SVM	http://www.imtech.res.in/raghava/sirnapred	2280		0.68	0.66	0.10
8	[40].	SVM	http://predictor.nchu.edu.tw/siPRED/	2431		0.77	0.53	0.09
9	[41]	Linear	http://biodev.extra.cea.fr/DSIR	2182		0.67	NA	Server not working
10	[42]	SVM	NA	2431		0.80	0.71	Server not available

*Pearson Correlation Coefficient (PCC) is the correlation between experimental and predicted viral siRNA efficacy.

^1^Performance on n-fold training dataset of the study.

^2^Performance on test data set of the study.

#V^345^ is the validation dataset of experimental viral siRNA.

The siRNA efficacy prediction methods developed using heterogeneous siRNA datasets are listed in Table 3. Their correlation performance during cross validation on the training set varied between 0.46 and 0.56 and for the test set their PCC was between 0.40 and 0.52 which shows the performance of these methods is almost similar. However, no siRNA efficacy prediction web server based on heterogeneous siRNA datasets was presently available/working except our ‘siRNApred’ which showed maximum PCC of 0.10 on V^345^ viral siRNA dataset.

The siRNA efficacy prediction algorithms developed using homogeneous siRNA datasets are given in Table 4. Their PCCs ranged from 0.66 to 0.85 on training during 10-fold cross validation and 0.55 to 0.71 on validation datasets. However on viral siRNA dataset V^345^ their performance dropped drastically in the range of 0.05 to 0.18. These results showed that the mammalian siRNA efficacy prediction methods developed using the homogeneous datasets performed better than those developed using the heterogeneous datasets. However, all such general mammalian siRNA efficacy prediction methods did not perform appropriately in predicting viral siRNA inhibition.

### Performance evaluation using Leave one out cross validation (LOOCV)

We also checked the performance of our method using LOOCV technique by employing earlier mentioned siRNA features (Table 5). Here also the performance was increased while using mono to penta nucleotide frequency and further using hybrid features. The performance is improved modestly in comparison to 10-fold cross validation. Using this technique we achieved a maximum correlation of 0.58 on the training dataset **T**^**1380**^ and **0.55** on the validation dataset **V**^**345**^ using the hybrid model combining hybrid nucleotide frequency, binary and thermodynamic features.

**Table 5:** Performance of the SVM models using leave one out cross validation (LOOCV) method

**Predictive model no.**	**siRNA features**		**No. of siRNA features**	Pearson correlation coefficient*	
				**Training (T**^**1380**^**)**	**Validation (V**^**345**^**)**
**1**		Mononucleotide frequency	4	0.32	0.29
**2**		Dinucleotide frequency	16	0.36	0.32
**3**		Trinucleotide frequency	64	0.45	0.41
**4**		Tetranucleotide frequency	256	0.48	0.44
**5**		Pentanucleotide frequency	1024	0.52	0.48
**6**		Binary	76	0.26	0.14
**7**		Thermodynamic features	21	0.29	0.24
**8**		Secondary structure	28	0.10	0.06
**9**		1 + 2 + 3 + 4 + 5	1364	0.52	0.49
**10**		6 + 9	1440	0.54	0.51
**11**		**6 + 7 + 9**	**1461**	**0.58**	**0.55**
**12**		6 + 7 + 8 + 9	1489	0.58	0.54

*Pearson Correlation Coefficient (PCC) is the correlation between experimental and predicted viral siRNA efficacy.

# T^1380^ is the training dataset of experimental viral siRNA. Predictive Models 1-8 were developed on individual siRNA features while models 9-12 were based on hybrid siRNA features.

### Performance evaluation using Leave one virus out cross validation (LOVOCV)

To further check our SVM based method on the best performing features combining hybrid composition (mono to penta nucleotide frequency), binary and thermo properties for each virus in the 1725 viral siRNA dataset, we used LOVOCV method. The results are shown in Table 6. In the LOVOCV method, 252 siRNAs from Influenza A Virus were excluded and the model trained with remaining 1473 siRNAs from 36 viruses. It showed a PCC of 0.48 and 0.46 on the training and validation dataset respectively. Similar performance PCC is observed for different viruses like Influenza A Virus (0.48 and 0.46), HCV (0.51 and 0.44), SARS (0.53 and 0.48), Measles Virus (0.56 and 0.51), JEV (0.58 and 0.51) etc. on the training and validation dataset respectively. Overall, the training dataset performance during 10-fold cross validation ranged from PCC value of a minimum 0.43 to a maximum 0.58 with an average 0.54. While the validation performance ranged from PCC 0.40 to 0.53 with an average 0.48.

**Table 6:** Performance of the SVM model for each virus in the 1725 viral siRNA dataset using leave one virus out cross validation (LOVOCV) method

**S.no.**	**Virus**	**No. of siRNA**		Pearson correlation coefficient*	
		**Training**	**Validation**	** Training#**	** Validation**
1	Influenza A Virus	1473	252	0.48	0.46
2	Human Papillomavirus	1513	212	0.43	0.40
3	John Cunningham Virus	1517	208	0.43	0.41
4	Respiratory Syncytial Virus	1577	148	0.45	0.41
5	Human Immunodeficiency Virus	1590	135	0.46	0.42
6	Metapneumovirus	1610	115	0.48	0.43
7	Hepatitis B Virus	1638	87	0.51	0.45
8	Hepatitis C Virus	1645	80	0.51	0.44
9	Ebola Zaire Virus	1652	73	0.49	0.43
10	Human Coxsackievirus	1653	72	0.50	0.47
11	West Nile Virus	1685	40	0.51	0.47
12	Bovine Papillomavirus	1689	36	0.52	0.48
13	Influenza B Virus	1689	36	0.52	0.46
14	SARS Coronavirus	1691	34	0.53	0.48
15	Herpes Simplex Virus	1704	21	0.54	0.48
16	Human Rhinovirus	1704	21	0.54	0.46
17	Orthopoxvirus	1705	20	0.55	0.49
18	Measles Virus	1709	16	0.56	0.51
19	Hepatitis Delta Virus	1710	15	0.56	0.51
20	Reovirus	1712	13	0.56	0.51
21	African Swine Fever Virus	1714	11	0.55	0.49
22	Dengue Virus	1714	11	0.56	0.49
23	Hazara Nairovirus	1714	11	0.56	0.49
24	Enterovirus	1717	8	0.56	0.50
25	Epstein-Barr Virus	1719	6	0.56	0.52
26	Hepatitis A Virus	1719	6	0.56	0.51
27	Human Metapneumovirus	1719	6	0.58	0.51
28	Hepatitis E Virus	1720	5	0.58	0.53
29	Japanese Encephalitis Virus	1720	5	0.58	0.51
30	St. Louis Encephalitis	1720	5	0.58	0.53
31	Junin Virus	1721	4	0.58	0.52
32	Yellow Fever Virus	1721	4	0.58	0.52
33	Lassa Virus	1722	3	0.58	0.52
34	Rotavirus	1723	2	0.58	0.53
35	Sendai Virus	1723	2	0.58	0.51
36	Marburg Virus	1724	1	0.58	0.52
37	Polio Virus	1724	1	0.58	0.53

*Pearson Correlation Coefficient (PCC) is the correlation between experimental and predicted viral siRNA efficacy.

#During 10-fold cross validation training, best performing viral siRNA sequence feature combining composition (mono to penta nucleotide frequency), binary and thermo features: 6 + 7 + 9) were used.

### Viral siRNA target conservation

Results of the viral siRNA target site conservation among reference viral genome sequences is provided in the Additional file 1: Figure S2. The analysis shows the number of nucleotide differences or mismatches (0, 1, 2, 3, 4 and >4) between each siRNA and the respective reference viral genome sequences in the alignment using Align0 algorithm. Overall percentages of the 0, 1, 2, 3, 4 and >4 mismatches were 36.75, 15.64, 9.01, 8.08, 7.76 and 23.68% respectively as shown in Additional file 1: Figure S2 (a). We have also checked the conservation of 322 highly effective siRNAs with inhibition above 80% and an equal number of least effective siRNAs with inhibition less than 4.0% from the main dataset. The effective and ineffective siRNAs had similar number of 1, 2, 3 and 4 mismatches with more differences for 0 and >4 mismatches as shown in Additional file 1: Figure S2 (b).

### Webserver

**Figure 1:**
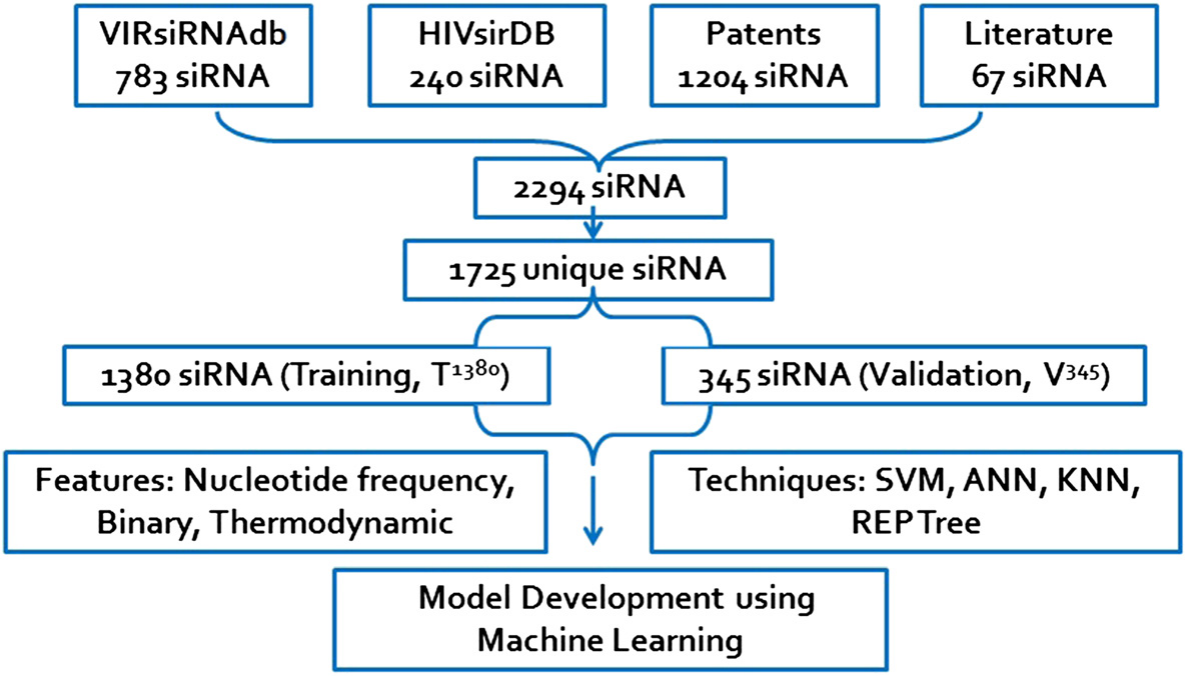
Workflow of the VIRsiRNApred model development.

**Figure 2:**
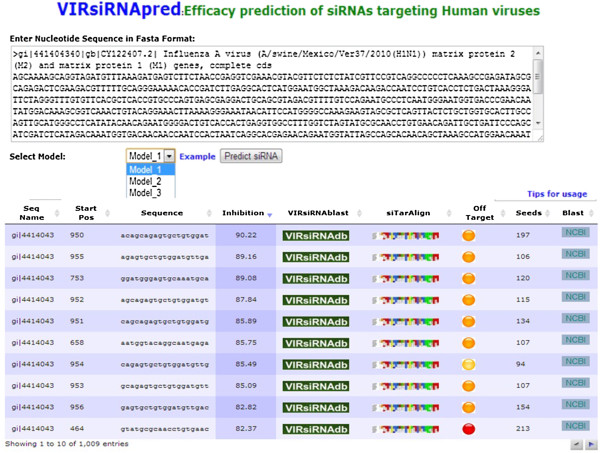
Web server and its functionality (top) submit page (bottom) result output.

The web server is freely available via the url http://crdd.osdd.net/servers/virsirnapred. The strategy used to develop VIRsiRNApred models is shown in Figure 1. Besides, overview of VIRsiRNApred web server functionality including input and output interfaces are shown in Figure 2. To predict the best performing siRNAs to silence a specific viral gene, the user needs to paste a fasta sequence of the corresponding mRNA/gene region and choose the desired model and click submit. The sequence should not contain non-nucleotide characters or symbols. The output shows the recursive siRNAs chopped from the mRNA & their inhibition. We have also displayed useful links pointing to BLAST, alignment and off-target score for each siRNA. The user can sort the results in increasing/decreasing order. The output also shows graph depicting the inhibition and off-target scores in a pictorial manner (Figure 2).

### Analysis tools

The analysis tools help the user to select the best antiviral siRNAs for the desired gene/region. First, the user needs to sort the siRNAs with “inhibition” and choose the siRNAs with high inhibition values. Then the user needs to look for the off-target using the two provided options: a) the user should select the siRNAs with least number of seeds/off-targets which are also shown pictorially by different colours as green (minimum), yellow (medium) and red (maximum) and b) the user can BLAST each siRNA sequence against human genome to ascertain that it does not affect other human genes. Besides, VIRsiRNAdb-BLAST helps the user to find similar siRNAs reported in the VIRsiRNAdb database.

Subsequently, to check the conservation of siRNA among different viral strains, our web server provides the “**siTarConserve**” tool. In this tool, user provided siRNA sequence is matched against selected reference viral genome sequences. There are options to search for conservation using individual virus as well as its family. The option to analyse conserved siRNA target regions using BLAST and Smith-Waterman algorithm has also been incoporated. This tool will be useful in selecting those siRNAs which are highly conserved in reference viral strains. Also while predicting siRNA efficacy, the user can check for siRNA conservation by clicking on the siTarConserve link provided against each siRNA on the prediction result page.

## Discussion

Researchers working in the field of siRNA based therapeutics for viruses have generated a vast amount of data over the years. However, bioinformatics resources in the field were lacking and there was no viral siRNA efficacy prediction method available. In this direction, we have recently developed a comprehensive viral siRNA database “VIRsiRNAdb” [48] and another “HIVsirDB” [49] exclusively for HIV. Now we have developed VIRsiRNApred -a viral siRNA efficacy prediction algorithm.

Although many mammalian siRNA prediction algorithms have been developed in the past [33], these methods either classify a siRNA as effective/non-effective [29] or predict the inhibition efficacy of a siRNA [31,32]. However, there is limited success in predicting siRNA efficacy due to limited size and diversity of available siRNA datasets [50].

Mammalian siRNA efficacy prediction methods were initially developed using siRNA tested under heterogeneous experimental conditions like Saetrom (581 siRNA), Shabalina (653 siRNA) or Holen (526 siRNA) etc. and achieved a PCC of 0.46-0.56 and subsequently methods were developed using siRNAs tested under homogenous experimental conditions like Heusken (2431 siRNA) and Katoch (702 siRNA) to achieve a PCC of 0.66-0.80. Though the mammalian siRNA prediction methods developed on homogenous siRNA datasets performed better than those developed on heterogeneous siRNA datasets, however all such methods did not perform well in predicting viral siRNA efficacy with PCC as low as 0.05-0.18.

We have developed our algorithm using a heterogeneous dataset of 1725 experimentally validated siRNA collected from 161 studies and targeting more than 200 mRNAs of over 40 diverse human viruses. Earlier Reynolds [23] has reported that 16.3% of randomly selected siRNAs have greater than 80% efficacy in targeting two mammalian genes (human cyclophilin B and firefly luciferase). Similarly, in 1725 viral siRNA dataset, only 17.1% were able to inhibit the virus above the 80% level despite majority of these siRNAs were designed using existing siRNA prediction methods before their testing in the wet-lab. This further verifies that the available mammalian siRNA efficacy prediction methods do not perform satisfactorily in predicting siRNAs targeting viruses.

Mammalian siRNA design rules are suitable for getting effective sequences for specific genes but they might sometimes prove unsuitable for selecting sequences for many other genes [51]. Therefore if these rules were used to design siRNAs for say, viral genes, many sequences might be wrongly selected as potential candidates. Even within mammalian siRNA prediction methods, the nucleotide positional preferences are not consistent (Additional file 1: Figure S1) with increase in heterogeneity of the data. Hence, designing functional siRNAs that target viral sequences is problematic because of their extraordinarily high genetic diversity. Therefore we need an algorithm to be developed on diverse viral siRNA dataset to tackle this issue.

However, in the past there have been attempts to predict viral siRNA. Naito et al. in 2006 published siVirus to classify a given viral sequence as siRNA based on earlier published guidelines of Ui-Tei et al., Reynolds et al. and Amarzguioui et al. for a few viruses [28]. Later, ElHefnawi in 2011 also provided a guideline to design siRNA against Influenza A virus using earlier published guidelines of Tuschl et al., Reynolds et al., Amarzguioui et al., Ui-Tei et al., and Hsieh et al., to select the siRNA [52]. However these methods just predict the viral siRNA as effective or non-effective based on earlier siRNA design rules. But they did not utilize any machine learning based methods which perform better than general siRNA design guidelines [33]. Moreover these methods were not developed using any experimental viral siRNA dataset and also do not predict quantitative efficacy of siRNA.

In the present study we made an effort to enhance the *in silico* prediction of highly effective human viral siRNA using manually curated datasets. Our model employed various siRNA sequence features particularly nucleotide frequencies, binary patterns, thermodynamic features, secondary structure and their hybrids as these parameters play a vital role in predicting efficient viral siRNAs. Mononucleotide composition can, for example, account for GC contents separately while higher nucleotide compositions can account for presence of any long GC stretch. Similarly trinucleotide composition can be useful in codon preference considerations. Studies have suggested that thermodynamic features of nucleotides at the beginning and at the end of the siRNA strands are important to their potency [53]. An increase in Pearson Correlation Coefficient (PCC) from 0.19 to 0.55 was observed using different approaches. The increase in performance could be attributed to the fact that as we increase the length of siRNA fragment (e.g., from 4 patterns in mono to 1024 patterns in pentanucleotide frequencies) more and more input information is available for processing not only in terms of composition but also other features like base stacking and positional effect to a significant extent. However secondary structural features of nucleotides showed minimal performance during training and were thus not included in the final model. Hence, the sequence frequencies combined with binary and thermodynamic features as hybrid approach we could achieve a maximum PCC of **0.58** during LOOCV. Thus, individual siRNA features provide marginal but cumulative increase in the probability of selecting a potent siRNA which is consistent with previous findings [32,33].

The LOVOCV method showed similar performance for most of the viruses; however Performance PCC improved with increasing number of siRNAs in the training dataset. Since viruses are known to be genetically diverse [54] some variation in performance is expected, however, the hybrid features allow different siRNA features to complement each other during the training process, which eventually makes the efficacy prediction better than when the features are used individually. LOVOCV performances are similar to that of PCC (0.55 and 0.50) of 10-fold cross validation of T^1380^ training and V^345^ validation datasets using best hybrid siRNA features reported in Table 1. Despite exclusion of individual virus siRNAs from the training dataset, SVM model predicts siRNA appropriately for that particular virus. Therefore, VIRsiRNApred would function as a general viral siRNA efficacy predictor even for viruses currently not included in the training and testing. Besides, we also checked the performance of general mammalian siRNA predictors (siRNApred and siExplorer) on the above datasets and their performance PCC ranged from 0.10 to 0.16 only. This again accentuate that VIRsiRNApred perform competently for prediction viral siRNA efficacy.

Since viruses are known to be genetically diverse [54], some researchers have used conserved target sites to design siRNAs [28]. ElHefnawi has used this approach to design siRNAs against the influenza A virus [52]. Similarly Rosales et al. predicted siRNAs against NS4B and NS5 of Dengue virus [55] and Raza et al. predicted siRNAs against HA and NA genes of Influenza virus A based on sequence conservation and alignment [56]. Importance of selection of conserved regions targeted by siRNA in HIV-1 has been discussed by Naito et al. [28,57].

In the overall conservation analysis of 1725 viral siRNAs with their respective reference genomes only around 37% were fully conserved (0 mismatch). It could be because of high viral genome heterogeneity. Besides that the researchers often target a particular viral strain and the target site may not be conserved among all other strains. However conservation analysis of highly and least effective siRNAs showed that number highly effective siRNAs are more in the fully conserved regions (0 mismatch) whereas the number of ineffective siRNAs is more in the least conserved regions (>4 mismatches). This suggests that siRNAs selected in the conserved regions tend to be more effective. However siRNAs designed from highly conserved virus genome regions as available in our experimental dataset display all ranges of siRNA inhibition efficacies ranging from 0-100%. This implies that not all siRNAs chosen from conserved regions are highly effective as many other factors like nucleotide frequencies, binary and thermodynamics features etc. contribute to the efficacy of a siRNA besides conservation as used in the VIRsiRNApred development. Nevertheless, selecting potential siRNAs from conserved genome regions is advantageous as it will target multiple viral strains.

This regression model can be useful in selecting siRNA molecules targeting viral genes for therapeutic purpose. Using our web-server researchers can target any given viral mRNA and get a list of highly performing siRNAs which have a greater chance to fully degrade the viral gene. In addition the ‘VIRsiRNApred’ server also offers an updated list of the experimentally validated siRNAs along with their numerical inhibition values reported in scientific literature that have been used against diverse types of viral genes. Also our web-server offers predicted siRNAs against important genes of HIV, Influenza, HCV, HBV and SARS. As siRNAs have been used against many viruses targeting a variety of genes and a few are already in different stages of clinical trials so they can serve as useful tools to develop potential antiviral drugs.

Currently the data availability for viral siRNAs comes from a wide range of studies due to which the overall performance of the model is affected. As more homogeneous data is generated from high-throughput studies, we will be interested in updating our prediction models in accordance with the new information to further improve the predictive power of our algorithm.

## Conclusions

In this study we have reported the first viral siRNA efficacy prediction algorithm developed on experimentally verified viral siRNAs targeting as many as 37 diverse human viruses since existing general mammalian siRNA prediction methods are not able to effectively predict viral siRNA activity. VIRsiRNApred web-server will be helpful to select potent virus inhibiting siRNAs that can increase the knock down success rate and thus shorten the validation time in the development of antiviral siRNA therapeutics.

## Abbreviations

RNAi: RNA interference; siRNA: Small interfering RNA; VIRsiRNApred: Viral siRNA predictor; LOOCV: Leave on out cross validation; LOVOCV: Leave on virus out cross validation; SVM: Support vector machine; RBF: Radial basis function; ANN: Artificial neural network; SNNS: Stuttgart neural network simulator; KNN: K-Nearest Neighbour; REP: Reduced error pruning; V345: Validation dataset; T1380: Training dataset; siTarConserve: siRNA Target conservation tool.

## Competing interests

The authors declare that they have no competing interests.

## Authors’ contributions

MK conceived the approach, helped in analysis and interpretation of data, gave overall supervision to the project. MK, AQ wrote the manuscript. AQ, NT collected the data, implemented machine learning software and developed the web server. All of the authors read and approved the final manuscript.

## Supplementary Material

Additional file 1: Figure S1 Comparison of highly and least effective siRNAs using two sample logo. **Figure S2.** Viral siRNA target conservation. Click here for file
