# Supplementary material for: VIRsiRNApred: a web server for predicting inhibition efficacy of siRNAs targeting human viruses
Source: J Transl Med. 2013 Dec 11;11:305. doi: 10.1186/1479-5876-11-305 (PMC3878835; doi:10.1186/1479-5876-11-305)
Supplement: Additional file 1: Figure S1 — Comparison of highly and least effective siRNAs using two sample logo. Figure S2. Viral siRNA target conservation. [file 1479-5876-11-305-S1.doc]

**Additional file 1**

**Figure S1 Comparison of highly and least effective siRNAs using two sample logo.**

**Figure S2 Viral siRNA target conservation.**

**(a) Huesken 2431**


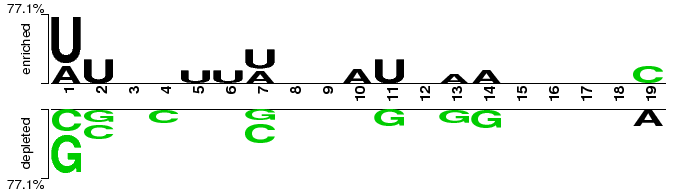


**(b) Saetrom 581**


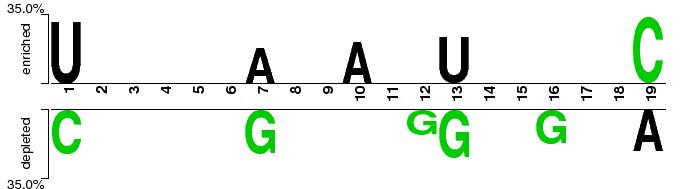


**(c) VIRsiRNA 1725**


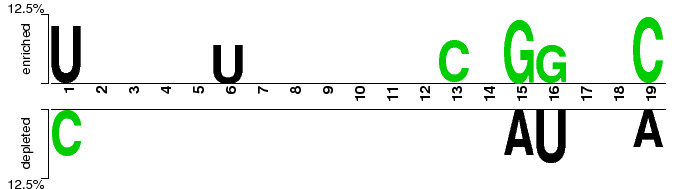


**Figure S1** Two sample logos of highly effective siRNAs vs least effective siRNAs in (a) Huesken 2431 (b) Saetrom 581 and (c) VIRsiRNA 1725.

In order to compare the positional effect of different nucleotides in mammalian siRNA datasets along with our viral siRNA dataset, we used two sample logo server (<http://www.twosamplelogo.org/cgi-bin/tsl/tsl.cgi>) as shown in Figure S1. Here we selected the best performing siRNAs with efficacy value greater than or equal to 80% as positive and least active siRNAs with efficacy value less than or equal to 25% as negative by sorting the data in increasing/decreasing values of percent inhibition in each case. Antisense siRNA sequences were used from the three datasets of Heusken (Huesken et al. 2005), Saetrom (Saetrom 2004) and our Viral siRNA dataset. The last two nucleotides of Heusken dataset (21mer) were chopped to make their length equal to 19mer for comparison with other datasets. Equal numbers of positive as well as negative siRNA sequences were selected from each dataset which were falling in the defined range. From Huesken dataset, 70 siRNAs were selected having siRNA efficacy value above 80% for positive and below 25% for negative sequences. Similarly from Saetrom dataset, 40 siRNAs were selected of which positive have efficacy value above 95% and negative sequences have efficacy below 24%. We have also selected 322 siRNAs having efficacy above 80% and below 4% for positive and negative siRNA respectively from viral siRNA dataset.  The Huesken and Saetrom mammalian siRNA datasets showed some similarity in the preference of nucleotides at positions 1 (U/C), 7 (A/G), 10 (A/-), and 19 (C/A). However in case of viral siRNA data, only positions 1 and 19 showed similarity with the above datasets where the nucleotides (U/C) and (C/A) were enriched at the ends. Also the two sample logos reveal that the preferred residues were enriched 77% in case of Huesken, 35% for Saetrom and 12.5% for viral siRNA. This may be due to the more diverse and heterogeneous nature of the viral siRNA dataset compared to Huesken and Saetrom siRNA datasets. These results also confirm the fact that binary (positional) feature used during machine learning does not work effectively in case of viral siRNA but comparatively shows good correlation in case of mammalian homogeneous siRNA data.


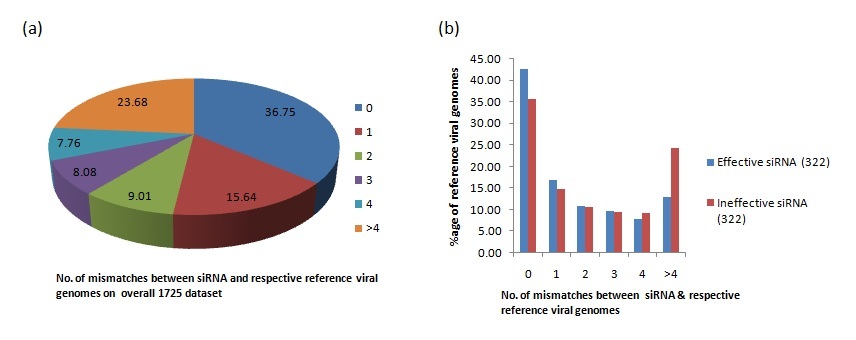


**Figure S2** (a) Percentage of mismatches between siRNA and respective reference viral genomes on overall 1725 viral siRNA. (b) Conservation analysis of 322 highly effective siRNAs with inhibition above 80% and an equal number of least effective siRNAs.
